# Supplementary material for: Adapting clinical chemistry plasma as a source for liquid biopsies
Source: eLife. 2026 May 1;14:RP108708. doi: 10.7554/eLife.108708 (PMC13134851; doi:10.7554/eLife.108708)
Supplement: Supplementary file 3. [file elife-108708-supp3.pdf]

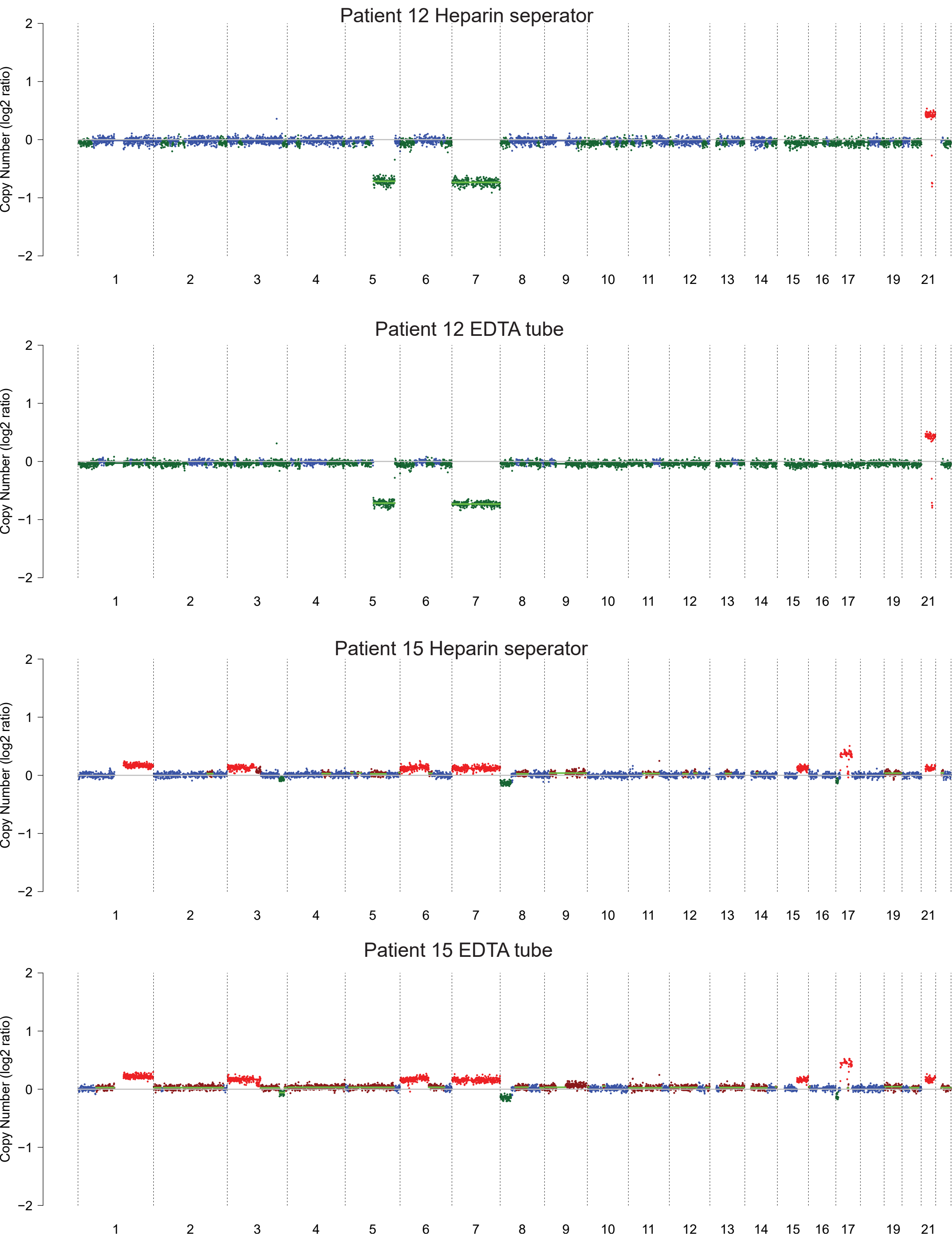
Patient 12 Heparin seperator
Patient 12 EDTA tube
Patient 15 Heparin seperator
Patient 15 EDTA tube
Copy Number (log2 ratio)
2
1
0
−1
−2
1 2 3 4 5 6 7 8 9 10 11 12 13 14 15 16 17 19 21

Patient 19 Heparin seperator

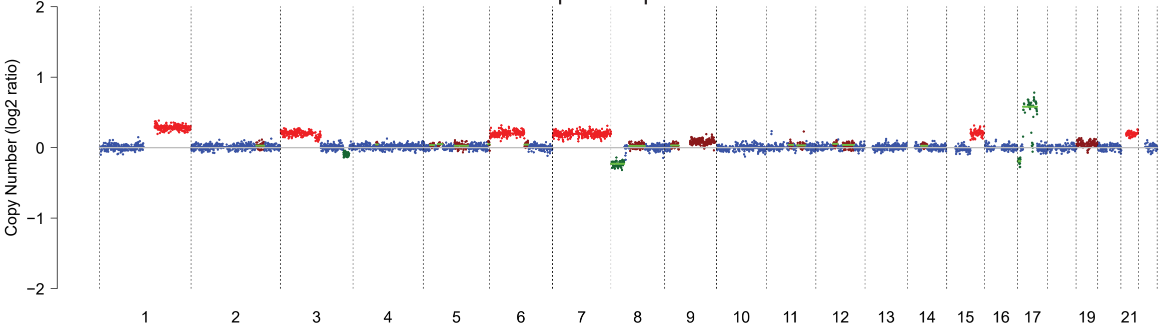

Patient 19 EDTA tube

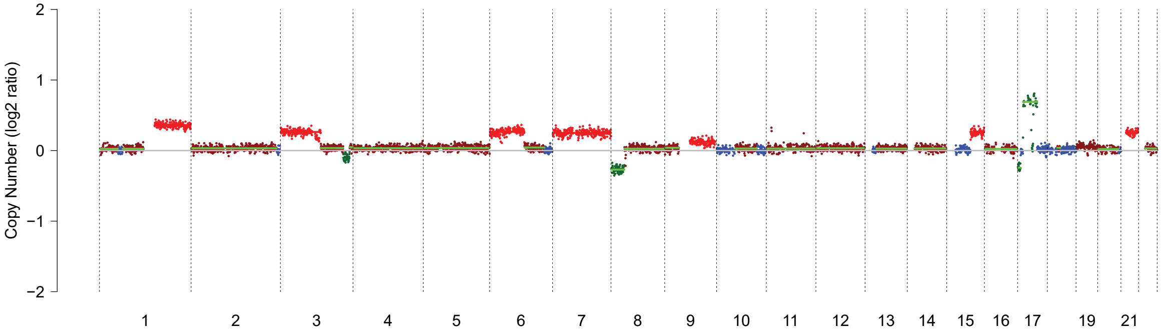

Patient 22 Heparin seperator

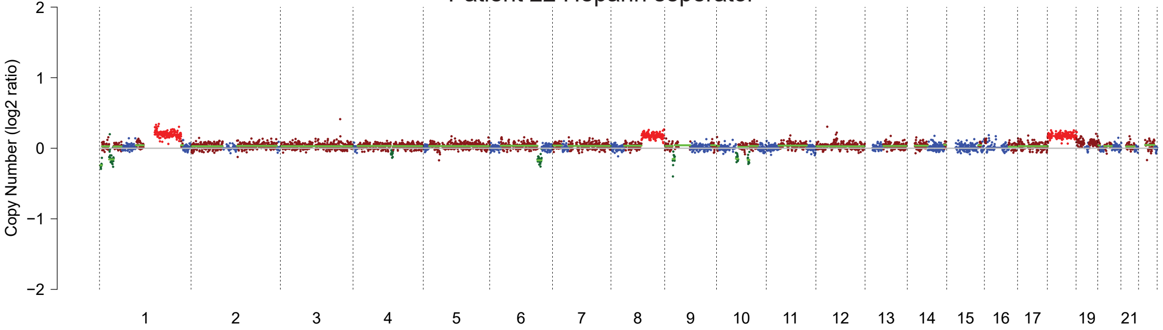

Patient 22 EDTA tube

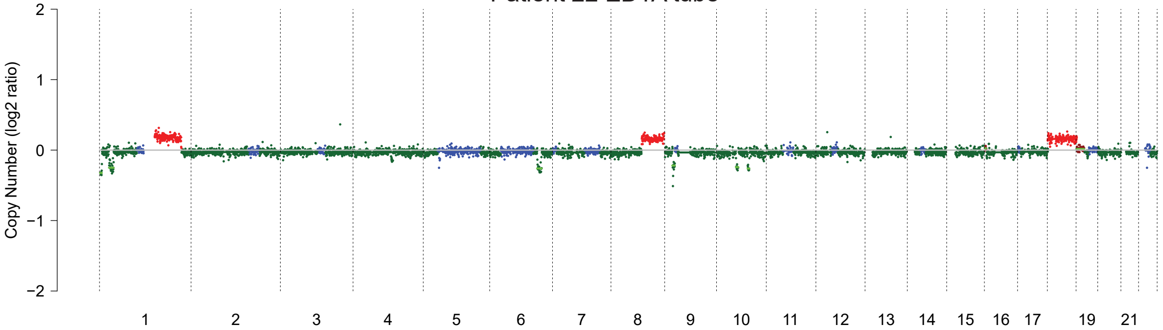

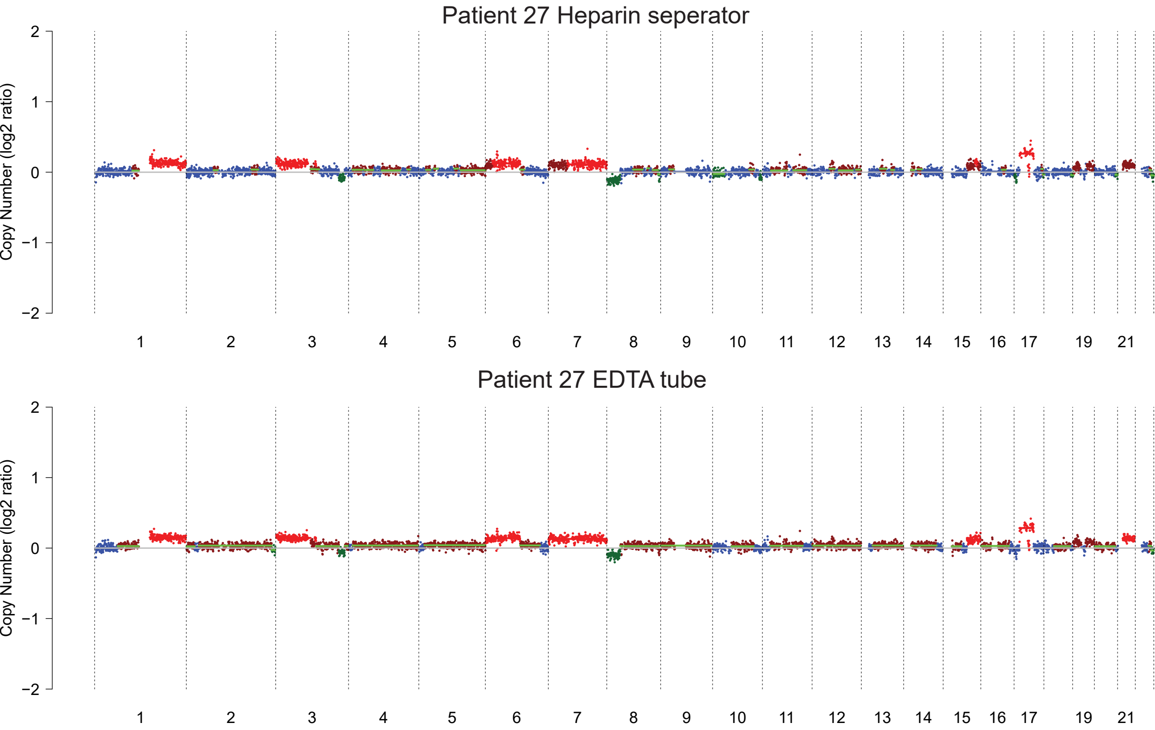

Patient 27 Heparin seperator
Copy Number (log2 ratio)
2
1
0
−1
−2
1
2
3
4
5
6
7
8
9
10
11
12
13
14
15
16
17
19
21
Patient 27 EDTA tube
Copy Number (log2 ratio)
2
1
0
−1
−2
1
2
3
4
5
6
7
8
9
10
11
12
13
14
15
16
17
19
21
